# A dosimetric and treatment efficiency evaluation of stereotactic body radiation therapy for peripheral lung cancer using flattening filter free beams

**DOI:** 10.18632/oncotarget.12071

**Published:** 2016-09-16

**Authors:** Ji-Yong Zhang, Jia-Yang Lu, Li-Li Wu, Dan-Li Hong, Chang-chun Ma, Xun Peng, Zhi-Xiong Lin

**Affiliations:** ^1^ Department of Radiation Oncology, Cancer Hospital of Shantou University Medical College, Shantou 515031, China; ^2^ Department of Endocrinology, The First Affiliated Hospital of Shantou University Medical College, Shantou 515031, China

**Keywords:** dosimetric benefits, stereotactic body radiation therapy, flattening filter free beams, lung cancer

## Abstract

To investigate potential dosimetric benefits and treatment efficiency of dynamic conformal arc therapy (DCA), intensity modulated radiation therapy (IMRT), and double partial arcs Rapidarc (RA) techniques in the treatment of early-stage peripheral lung cancer using stereotactic body radiotherapy (SBRT) with flattening filter free (FFF) beams. Twenty early-stage peripheral lung cancer patients were selected. For each patient, DCA, IMRT and RA plans were created to meet Radiation Therapy Oncology Group (RTOG) 0915 objectives with 48 Gy covering 95% of the planning target volume (PTV) in 4 fractions. PTV coverage, organs at risk (OARs) doses, planning time, monitor units (MU) and treatment time were evaluated. RA was significantly better than DCA for PTV coverage. RA provided a lower V_32Gy_ to chest wall and less V_20Gy_ to lung over those of DCA and IMRT. For other OARs, there is no significant difference among all three techniques. DCA plans showed significantly less planning time, shorter treatment time and lower MU number than those of RA and IMRT. RA provides a superior dosimetric benefit to DCA and IMRT in the treatment of early-stage lung cancer using SBRT with FFF beams. Considering the MU number, planning time and treatment efficiency, DCA technique is an effective treatment strategy.

## INTRODUCTION

Lung cancer is the major reason of cancer death among males [1]. For females, lung cancer is the primary cause of cancer death in more developed countries, and the lung cancer incidence rates in Chinese women are 204 cases per one million [1]. Non-small-cell lung cancer (NSCLC) takes up more than 85% of all lung cancer [2]. For early-stage NSCLC patient, surgery is still the considerable choice in the treatment [3, 4]. Nevertheless, not all early-stage NSCLC patients are suited for surgery because of advanced age, or patients refusing surgical treatment. Stereotactic body radiotherapy (SBRT) is an alternative method for these patients.

SBRT is a method of radiation therapy that delivers a high radiation dose in a few fractions [5]. This hypofractionation technique has showed better local control rates when compared to the conventional fractionated radiotherapy [6]. Previous clinical researches have shown positive results on treating the early-stage NSCLC patient with SBRT technique [7–9]. In the past, three-dimensional conformal radiation therapy (3DCRT) and intensity-modulated radiation therapy (IMRT) are the two most common techniques for SBRT. Recently, advanced radiotherapy delivery techniques, such as Rapidarc (RA) is becoming a better method for the delivery of SBRT. RA is a form of volumetric modulated arc therapy (VMAT) that has been shown to improve the treatment efficiency [3].

Like RA, dynamic conformal arc therapy (DCA) is a delivery technique which the multileaf collimator (MLC) dynamically shapes the target with a rotating gantry [5, 10]; however, DCA technique does not involve the optimization process. After generating the MLC apertures, we directly carry out dose calculation. Although DCA only shapes the MLCs to the target, it still can provide a conformal dose distribution [11].

In this study, the early-stage peripheral NSCLC patients were selected, and we applied the DCA, IMRT and double-arc Rapidarc techniques for the patients with flattening filter free (FFF) beams, the dose constraints followed the radiation therapy oncology group (RTOG) 0915 protocol [12]. The purpose of this study was to investigate the potential dosimetric benefits and treatment efficiency of the DCA, IMRT and RA techniques in the treatment of inoperable early-stage NSCLC using SBRT with FFF beams.

## RESULTS

**Figure 1:**
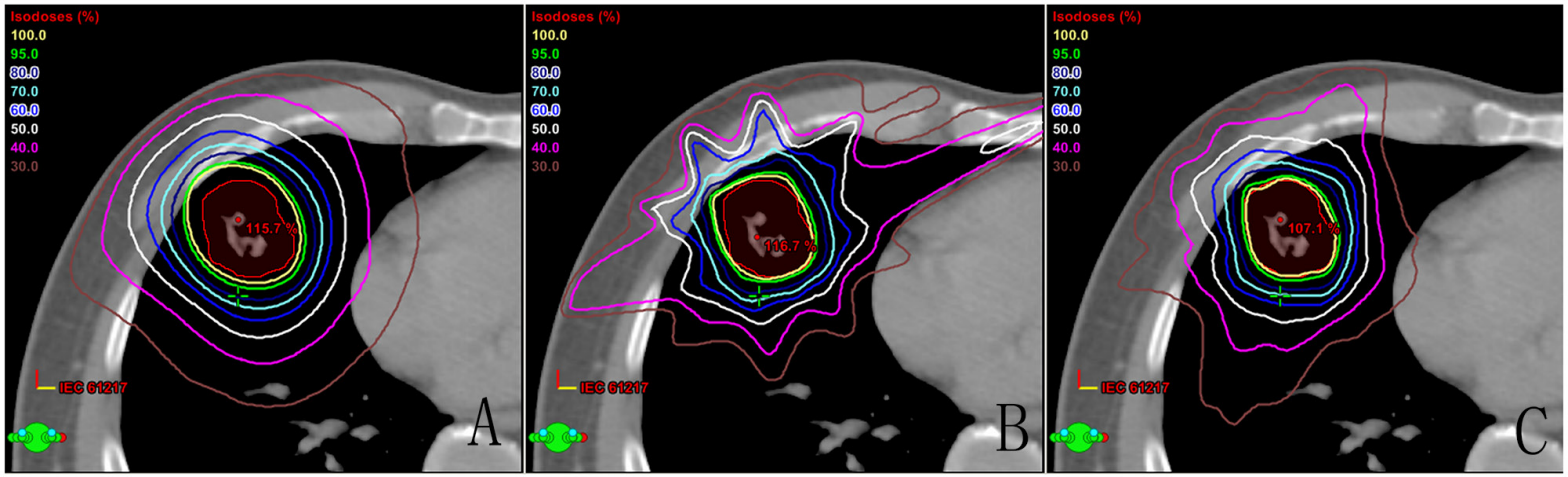
Comparison of dose distribution for each of the three techniques: A. dynamic conformal arc therapy, B. intensity modulated radiation therapy, C. double partial arcs Rapidarc

One example of dose distribution was shown in Figure 1 for each treatment technique. Dose volume histogram (DVH) was created for each treatment plan and utilized to assess the PTV coverage and dose to OARs. All DCA, IMRT and RA plans met the RTOG 0915 protocol criteria for the PTV coverage.

### PTV coverage

**Figure 2:**
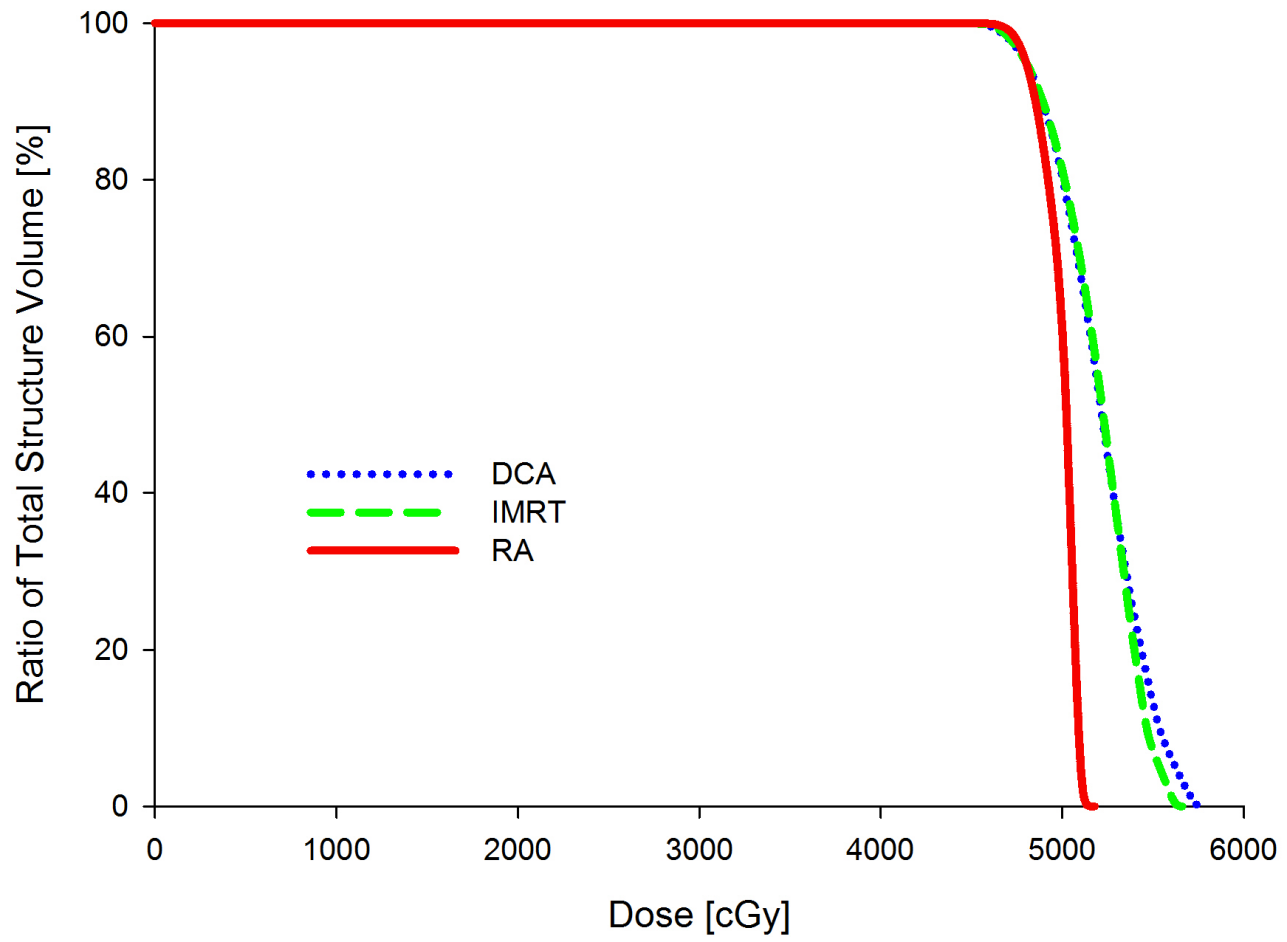
DVHs of PTV for dynamic conformal arc therapy (DCA), intensity modulated radiation therapy (IMRT) and double partial arcs Rapidarc (RA)

Figure 2 shows the DVH for the PTV with the DCA, IMRT and RA plans. Comparisons of the treatment planning techniques for the PTV were summarized in Table 1. Between DCA and IMRT, no statistical difference was observed in terms of D_98%_, D_50%_, D_2%_, V_90%_, V_95%_ and V_105%_. Similar to DCA and IMRT, between IMRT and RA, statistical significant results were not observed. There was a significant difference between DCA and RA for D_2%_ (*p*<0.001). RA displayed a slightly better PTV coverage than DCA.

**Table 1:** Summary of the PTV coverage for DCA, IMRT and RA techniques

	DCA	IMRT	RA	*P* Value		
	Mean(Range)	Mean(Range)	Mean(Range)	DCA vs IMRT	DCA vs RA	IMRT vs RA
D_2%_(cGy)	5505.86(5338.93-5703.29)	5549.79 (5243.5-5873.48)	5202.44 (4999.8-5424.88)	0.554	0.000	0.472
D_98%_(cGy)	4702.74(4667.81-4730.1)	4681.74 (4584.37-4725.8)	4726.25 (4691.8-4755.8)	0.190	0.017	0.793
D_50%_(cGy)	5177.64(5129.5-5279.85)	5259.60 (5103.1-5587.32)	5081.66 (4916.4-5247.98)	0.117	0.010	0.338
V_90%_(%)	99.99(99.93-100)	99.91(99.37-100)	100(99.96-100)	0.225	0.714	0.511
V_95%_(%)	99.68(99.27-100)	99.44(98.2-99.9)	99.87(99.54-100)	0.261	0.072	0.910
V_100%_(%)*	95	95	95	-	-	-
V_105%_(%)	72.95(68.2-80.33)	77.05(63.4-86.79)	54.63(0-80.57)	0.159	0.046	0.744

* Due to all plans were normalized to 100% of prescribed dose covering 95% of the PTV, the value of the V100%(%) for each plan was 95.

### Dose to the OARs

Table 2 shows the dosimetric outcomes of the OARs for DCA, IMRT and RA techniques. No statistical difference was observed among DCA, IMRT and RA treatment plans for the Dmax of lung, heart, spinal cord, bronchial tree, esophagus, skin and great vessels. For the maximum volume of these critical organs, there is no significant difference among three techniques except the chest wall. The low-dose spillage of normal tissue was evaluated by R_50%_ and D_2cm_. The R_50%_ of RA technique was significantly lower than those of DCA (*p*<0.001) and IMRT (*p*<0.001) techniques; the DCA technique was higher than IMRT technique for R_50%_. The D_2cm_ of RA technique was also lower than those of DCA and IMRT techniques, and IMRT technique was better than the DCA technique for D_2cm_.

**Table 2:** Summary of the OARs doses for DCA, IMRT and RA techniques

	DCA	IMRT	RA	*P* Value		
	Mean(Range)	Mean(Range)	Mean(Range)	DCA vs IMRT	DCA vs RA	IMRT vs RA
Lung						
V_20Gy_(%)	6.38(3.53-10.49)	5.14(2.23-8.66)	4.34(1.86-8.72)	0.246	0.064	0.096
V_5Gy_(%)	14.2(8.68-21.26)	14.72(8.37-19.56)	13.88(8.09-20.99)	0.789	0.886	0.68
D_mean_(cGy)	394.99(215.6-560.7)	330.86(183-457.4)	337.09(171.8-480.8)	0.298	0.376	0.912
Spinal Cord						
Dmax(cGy)	890.57(49.4-1822.2)	718.15(29.5-1479.1)	693.25(410.7-1127)	0.519	0.347	0.541
V_13.6Gy_(cc)	0.25(0-2)	0.00(0-0)	0.00(0-0)	0.286	0.284	0.284
Esophagus						
Dmax(cGy)	936.75(65.00-1913.00)	1152.51(652.00-2121.00)	1223.44(508.00-2436.00)	0.400	0.327	0.261
V_18.8Gy_(cc)	0.00(0.00-0.00)	0.01(0.00-0.12)	0.08(0.00-0.77)	0.343	0.343	0.217
Heart						
Dmax(cGy)	2162.99(54-5167.9)	2317.70(47.1-5705.7)	2210.99(54.8-5181)	0.852	0.950	0.916
V_28Gy_(cc)	3.15(0-23.49)	5.62(0-50)	1.79(0-13.06)	0.660	0.627	0.625
Great vessels						
Dmax(cGy)	1972.74(14.9-5149)	2180.43(11.7-5745.8)	2111.23(13.2-5388.6)	0.812	0.869	0.768
V_43Gy_(cc)	0.86(0-4.61)	0.33(0-1.71)	0.29(0-1.78)	0.406	0.369	0.511
Bronchial Tree						
Dmax(cGy)	2639.59(29.6-5395.1)	2629.86(24.1-5917)	2591.07(24.4-5461.1)	0.992	0.960	0.997
V_15.6Gy_(cc)	1.71(0-9.01)	1.27(0-5.19)	1.92(0-9.34)	0.703	0.872	0.899
Chest Wall						
Dmax(cGy)	4716.79(2947.6-5720.6)	4439.84(2734.5-5786.5)	3954.45(2195.2-5173.5)	0.545	0.121	0.295
V_32Gy_(cc)	14.42(0-30.57)	5.19(0-15.01)	3.53(0-12.13)	0.014	0.003	0.005
Skin						
Dmax(cGy)	2893.5(2107.3-5361.7)	3205.37(2203-4971.5)	2172.88(1439.5-4586.7)	0.470	0.145	0.203
V_33.2Gy_(cc)	0.65(0-5.16)	0.18(0-1.59)	0.07(0-0.7)	0.389	0.294	0.313
Other						
R_50%_	7.39(5.68-8.81)	6.04(4.67-8.54)	4.03(3.62-4.53)	0.013	0.000	0.000
D_2cm_(%)	79.24(70.13-84.94)	70.37(55.29-81.51)	52.52(45.36-59.14)	0.008	0.000	0.000

### PTV homogeneity and conformity

For the CI of PTV, there was a highly significant difference among the three radiotherapy techniques (Table 3). The CI of the RA technique was better than those of DCA technique (*p*<0.001) and IMRT technique (*p*<0.001), and the CI of the IMRT technique was also better than that of DCA technique (*p*<0.001). With respect to the HI of PTV, there was a significant difference between DCA technique and RA technique (*p*<0.001). However, there was no significant difference for IMRT technique compared to DCA technique and RA technique.

**Table 3:** Summary of technical features for DCA, IMRT and RA techniques

	DCA	IMRT	RA	*P* Value		
	Mean(Range)	Mean(Range)	Mean(Range)	DCA vs IMRT	DCA vs RA	IMRT vs RA
CI	1.45(1.24-1.61)	1.11(1.03-1.17)	1.01(0.99-1.08)	0.000	0.000	0.000
HI	0.15(0.12-0.19)	0.16(0.1-0.23)	0.09(0.05-0.14)	0.532	0.000	0.386
MU	1884.3(1734-2076)	3933.9(2824-5263)	2777.7(2167-3100)	0.000	0.000	0.000
TT(min)	1.83(1.73-1.95)	4.64(3.95-5.73)	2.37(2-2.55)	0.000	0.000	0.000
PT(min)	15.17(9-22)	73.67(50-93)	76.17(58-93)	0.000	0.000	0.755

*Abbreviations*: CI = conformity index; HI = homogeneity index; MU = monitor unit; TT = treatment time; PT = planning time.

### Monitor units and treatment time

The averaged total MU numbers of DCA plans were lowest, and IMRT plans had the highest MU number. There was a significant difference between all three techniques (*p*<0.001) (Table 3). Evaluation of treatment time revealed that the DCA plans were obviously faster than IMRT plans (*p*<0.001) and RA plans (*p*<0.001). The treatment time of DCA compared to those of IMRT and RA plans, decreased by 60.6% and 22.8%, respectively. For the planning time, the DCA plan was significantly quicker than IMRT and RA plans (*p*<0.001), there was no significant difference between IMRT and RA plans (*p*>0.05).

### Dosimetric verification of plans

The Delta4 phantom verification for all plans shows high passing rates between calculated and measured doses using a gamma analysis with a 10% dose threshold for 3% /3 mm criteria. The average passing rates for DCA plan, IMRT Plan and RA plan were 99.4 (range 98.5-100), 99.1(range 98.3-99.8) and 99.6 (range 99.1-100).

## DISCUSSION

Published studies have reported that SBRT technique improves both local control and overall survival for the inoperable early-stage NSCLC patients, with a 3-year survival rate of 45% and local control of 89% [13], and a 2-year survival rate of 74% [14]. The appliation of SBRT technique for the inoperable early-stage NSCLC patient is becoming a benchmarking of treatment. This study aimed to evaluate the dosimetric benefit and delivery efficiency of DCA, IMRT and RA plans with FFF beams for SBRT in inoperable early-stage NSCLC.

Several studies previously reported that VMAT plans were able to afford a superior conformal dose to the target than either DCA or IMRT plans [15, 16]. With regard to this study, the RA technique was able to provide a better PTV coverage than DCA and IMRT. For the D_2%_ (represents maximum dose), a significant difference was observed between RA and DCA in the PTV, and the D_2%_ of RA was lower than that of DCA. In addition, the D_98%_ (represents minimum dose) of RA was higher than that of either DCA or IMRT. All three techniques met the RTOG 0915 objective for the PTV for the prescription dose coverage. Concerning the dose conformity, our study showed that RA plans achieved a statistically better target conformity than DCA and IMRT plans, as has been demonstrated by Rauschenbach *et al*. [5] and Ong *et al* [17].

In this study, all of the patients had peripheral lung tumors; therefore, OARs dosimetric characteristic was determined by the tumor location. When the tumor is close to the OARs, there is a high probability that OARs constraints could not be met, such as the chest wall. Our results showed RA gave a lower maximum dose in the chest wall than other techniques. The comparison of the chest wall in this study did not agree with the results from the study by Liu *et al* [18]. In Liu *et al*, most of the patients had central lung lesion. In their comparison, no significant difference in maximum chest wall dose was observed between the RA and DCA. Furthermore, RA technique achieved a significant decrease in V_32Gy_ when compared with the DCA and IMRT. Recent clinical papers advised that if the volume of chest wall receiving 30 Gy was less than 30 cm^3^ it could decrease the risk of toxicity [19, 20]. In relation to the skin dose, RA plans provided better results. Moreover, DCA delivered a lower skin dose than IMRT, for superficial targets. In general, IMRT technique may cause the skin dose increased [21]. As shown in the results, RA did not provide a significant improvement in the V_20Gy_ and D_mean_ compared with DCA and IMRT. In addition, no difference was found in V_20Gy_ and D_mean_ to the lung between DCA and IMRT, only the RA achieved a little improvement. These results were verified in the studies by Ong *et al*. [17] and Bree *et al* [15]. For other critical organs such as heart, bronchial tree, esophagus, great vessels and spinal cord, the doses were well under the dose constraints and no significant difference was observed among the three techniques.

The patients were treated in supine position with arms on the body side in this study; because of the patients put their arms over their heads might bring a worse setup error than their arms naturally put the body side. This might lead to the patients have their arms in the middle of the irradiated area, when a continuous irradiating arc applied. The IMRT treatment could avoid irradiating the arms.

Regarding the treatment time, it is not included the imaging and patient setup, only measuring from the first beam-on to the last beam-off. The DCA showed a 60.6% treatment time reduction over IMRT, and the treatment time of DCA decreased by 22.8% compared with RA. Dickey *et al*. [16] and Morales-Paliza *et al.* [22] reported that DCA was able to provide reduced treatment time when compared with IMRT and VMAT, using a conventional 6 MV photon beams with a dose rate of 600 MU/min. Shorter treatment time could increase patient comfort, reducing patient motion and improving resource use. SBRT plan requires more time to verify patient position to reduce the setup errors using image-guidance radiotherapy (IGRT). Although IGRT could adjust the setup errors, the total treatment time would be extended. Hoogeman *et al*. [23] observed patient position would be changed during the treatment when the treatment time is more than 15 min. DCA plans were created by a subarc, the MLCs were dynamically conformed to the target with a margin, without involved the optimization process, and these will significantly decrease the planning time.

The accuracy of SBRT delivery has a rigorous requirement on the machine quality assurance (QA), before SBRT plans are delivery. Patient-specific QA was routinely carried out on the treatment machine, and the passing rates show good agreement between calculations and measurements [24].

In conclusion, RA provided a superior dosimetric benefit to DCA and IMRT in the treatment of early-stage NSCLC using SBRT with FFF beams. Furthermore, RA plans were able to acquire lower dose sparing to OARs when the OARs were closed to the target. However, considering the MU number, planning time and treatment efficiency, DCA technique is an effective treatment strategy.

## MATERIALS AND METHODS

### Ethics statement

The study protocol was approved by the Ethics Committee of the Cancer Hospital of Shantou University Medical College. Because this is not a treatment-based study, our institutional review board waived the need for written informed consent from the participants. The methods in the study were performed in accordance with the approved guidelines and regulations.

### Patient selection

From March 2014 to December 2014, twenty early stage peripheral NSCLC patients were selected for this study. There were sixteen (80%) men and four (20%) women with a mean age of 68.8 years (range 65-77 years). The planning target volume (PTV) varied in the range of 16.34 to 85.97 cm^3^. Patient characteristics are shown in Table 4.

**Table 4:** General patient information

Patient characteristics	N (%)
Patient	
Male	16 (80%)
Female	4 (20%)
Stage	
T1N0M0	9 (45%)
T1aN0M0	2 (10%)
T1bN0M0	3 (15%)
T2N0M0	4 (20%)
T2aN0M0	2 (10%)
Tumor location	
Left	14 (70%)
Right	6 (30%)
Age (years)	
Mean	68.8
Range	65-77
ITV Volume (cm^3^)	
Mean	14.16
Range	4.67-50.37
PTV Volume (cm^3^)	
Mean	35.63
Range	16.34-85.97

### Immobilization and target definition

All patients were scanned and treated in supine position with arms on the body side and performed free breathing. An overlay board and thermoplastic mask were used to immobilize the head, neck and shoulder regions. CT images were acquired using four-dimensional computed tomography (4DCT) (Brilliance CT Big Bore, Philips Medical Systems, Amsterdam, The Netherlands) with Varian real-time position management (RPM) respiratory gating system (Version 1.7.5, Varian Medical Systems, Inc., Palo Alto, CA). For each patient, a 3 mm slice thickness was used from the third cervical vertebra (C3) to the third lumbar vertebra (L3). The 4DCT images were transferred to the treatment planning system (TPS) to contour the target volumes and OARs.

The gross tumor volume (GTV) was delineated on all 10 phases of the 4DCT images, and the internal target volume (ITV) was created by combining the GTV from each respiratory phase. The PTV was then created by expanding a universal margin of 5 mm from the ITV. The target volumes and OARs (include lungs, heart, spinal cord, bronchial tree, esophagus, skin, great vessels, and chest wall) were contoured according to the RTOG 0915 protocol by a radiation oncologist.

### Treatment planning

Each patient plan was replanned using the DCA, IMRT and RA techniques, based on 6 MV FFF photons with a dose rate of 1400 MU/min. All plans were created to be delivered using the TrueBeam linac (Varian Medical Systems, Inc., Palo Alto, CA) with a 120-leaf Millennium multileaf collimator (MLCs). All plans were designed in Eclipse external beam planning system (Version 10.0.42, Varian Medical System, Inc., Palo Alto, CA). The final dose calculations were carried out with a grid of 2.5 mm using the Anisotropic Analytical Algorithm (AAA). All patients were treated with a prescribed dose of 48 Gy in four fractions covering 95% of the PTV, and 99% of the PTV was covered by least 90% of the prescription dose. The dose constraints of OARs were set to follow the dosimetric parameters of the RTOG 0915 protocol.

### DCA planning

All the DCA plans consisted of a single partial arc built from 340° to 179° for the tumor location in left lung or from 181° to 20° for the tumor location in right lung. A 30° collimator angle was employed for the arc. The partial arc utilized the isocenter, the isocenter point was positioned at the PTV center. The DCA technique utilized a single partial arc arrangement for the peripheral lesion in order to avoid the couch collision or gantry-patient collision. The “fit MLC to Structure” tool generated the dynamic MLC apertures for the PTV during the gantry rotation. The MLCs were fitted to the PTV using a 7 mm margin in the superior and inferior directions, and then a 5 mm margin was applied in the other directions.

### IMRT planning

IMRT plans were created with five coplanar beams, the beam angles were designed to protect the contralateral lung. The gantry angles were angulated by 210°, 280°, 330°, 20°, 160° and 200°, 350°, 35°, 80°, 150° for right and left lung, respectively. According to our optimization protocol, the parameters were set to achieve the RTOG dose constraints. IMRT fluence maps were optimized by an optimization tool from the dose constraints in the TPS, and then optimal fluence maps were transformed into the actual fluence maps by a leaf motion calculator. The sliding-window delivery type was selected for all fields with the leaf motion calculator.

### RA planning

For RA plans, since the tumor lesions can occur on the far side of the lung there is a risk of gantry-patient collision. To avoid the collision problem and to spare the contralateral lung, a technique utilizing partial arcs was used. In this study, all RA plans were designed with a double partial arcs Rapidarc (RA) technique. The RA plans were planned using two overlapping partial arcs with the gantry rotate from 181° to 20° or from 340° to 179°, according to the tumor location. Each partial arc contains 114 control points. The collimator angle was set at 30° or 330° in order to reduce the effect of tongue and groove leakage for all RA plans. The same optimization objectives were used for the PTV and OARs, as for the IMRT plan. Moreover, the “air cavity correction” and “inhomogeneity correction” features were set to “on”, which could enhance the dose calculation accuracy in the air cavities and various density tissues.

### Dosimetric analysis for the PTV and OARs

For the PTV, the values of D_98%_, D_2%_, D_50%_, V_90%_, V_95%_, V_100%_ and V_105%_ were reported. The conformity of the target was assessed by the conformity index (CI) [25]; CI values closer to 1.0 indicate a better conformation. The homogeneity index (HI) was used to evaluate the dose homogeneity of the PTV [26]; HI value nearer to zero indicates a more uniform dose distribution in the PTV. For the OARs, the serial tissues were evaluated by maximum point dose (D_max_) and maximum volume (V_xGy_), the serial tissues included heart, spinal cord, bronchial tree, esophagus, skin, great vessels, and chest wall; the parallel tissue (Lung) was appraised by the maximum volume. R_50%_ describes the ratio of the volume of 50% of the prescription dose isodose to the volume of the PTV. D_2cm_ represents the maximum dose (in % of dose prescribed) at 2 cm from the PTV in all directions.

### Dosimetric verification and treatment time

Each treatment plan was transferred to the Delta4 phantom (Scandidos, Uppsala, Sweden) for measuring the dose distributions. The measured dose distributions were compared with the calculated dose distributions to evaluate the dose consistency by a gamma analysis [24]. Treatment time was measured from the first beam-on to the last beam-off using a stopwatch.

### Statistical analysis

Dosimetric comparison between plans were evaluated using the Wilcoxon two-paired sample signed-ranks test. Significant differences were considered at the level of *p*<0.05. The SPSS v19.0 software (IBM, Chicago, IL) was employed for statistical data analysis.
